# Inhibition of Phospho-S6 Kinase, a Protein Involved in the Compensatory Adaptive Response, Increases the Efficacy of Paclitaxel in Reducing the Viability of Matrix-Attached Ovarian Cancer Cells

**DOI:** 10.1371/journal.pone.0155052

**Published:** 2016-05-05

**Authors:** Jeong In Choi, Sang Hi Park, Hee-Jin Lee, Dae Woo Lee, Hae Nam Lee

**Affiliations:** 1 Department of Obstetrics and Gynecology, Bucheon St. Mary’s Hospital, College of Medicine, Catholic University of Korea, Seoul, Republic of Korea; 2 Clinical Medicine Research Institute, Bucheon St. Mary’s Hospital, College of Medicine, Catholic University of Korea, Seoul, Republic of Korea; The University of Hong Kong, HONG KONG

## Abstract

**Objective:**

To identify the proteins involved the compensatory adaptive response to paclitaxel in ovarian cancer cells and to determine whether inhibition of the compensatory adaptive response increases the efficacy of paclitaxel in decreasing the viability of cancer cells.

**Methods:**

We used a reverse-phase protein array and western blot analysis to identify the proteins involved in the compensatory mechanism induced by paclitaxel in HeyA8 and SKOV3 ovarian cancer cells. We used a cell viability assay to examine whether inhibition of the proteins involved in the compensatory adaptive response influenced the effects of paclitaxel on cancer cell viability. All experiments were performed in three-dimensional cell cultures.

**Results:**

Paclitaxel induced the upregulation of pS6 (S240/S244) and pS6 (S235/S236) in HeyA8 and SKOV3 cells, and pPRAS40 (T246) in HeyA8 cells. BX795 and CCT128930 were chosen as inhibitors of pS6 (S240/S244), pS6 (S235/S236), and pPRAS40 (T246). BX795 and CCT128930 decreased pS6 (S240/S244) and pS6 (S235/S236) expression in HeyA8 and SKOV3 cells. However, pPRAS40 (T246) expression was inhibited only by BX795 and not by CCT128930 in HeyA8 cells. Compared with paclitaxel alone, addition of BX795 or CCT128930 to paclitaxel was more effective in decreasing the viability of HeyA8 and SKOV3 cells.

**Conclusion:**

Addition of BX795 or CCT128930 to inhibit pS6 (S240/S244) or pS6 (S235/S236) restricted the compensatory adaptive response to paclitaxel in HeyA8 and SKOV3 cells. These inhibitors increased the efficacy of paclitaxel in reducing cancer cell viability.

## Introduction

Because of its tolerable side effects and high response rate, paclitaxel is used as a standard drug in the treatment of ovarian cancer. However, the high recurrence and drug-resistance rates are major obstacles in the treatment of ovarian cancer. About 80% of patients with advanced-stage ovarian cancer who respond completely to first-line chemotherapy ultimately relapse [1]. Because of drug resistance, second-line chemotherapy, which is less effective than the initial drugs, is used for patients who experience recurrence within 6 months after treatment. The compensatory adaptive response to chemotherapy in ovarian cancer is one cause of drug resistance. Initiated by cancer cells, the compensatory adaptive response allows them to survive at drug therapy by reprogramming the cell signaling pathways and activating the survival mechanisms that lead to resistance. Combinations that include a second drug to inhibit the compensatory adaptive response may reduce the survival of cancer cells and increase the efficiency of cancer treatment.

Ribosomal S6 kinase is a protein kinase that is involved in signal transduction. S6 kinase is overexpressed and thought to play a tumor-promoting role in various cancers. [2–4]. Several lines of evidence suggest that S6 kinase plays an important role in the growth and dissemination of ovarian cancer [5]. A copy number gain in S6 kinase has been observed in human ovarian carcinomas [6, 7]. S6 kinase can also be activated via amplification of the PI3K p110α catalytic subunit or AKT, mutation of the PI3K p85α regulatory subunit, or loss of PTEN, which are frequently observed in ovarian cancer [8, 9].

Normal epithelial cells form well-organized polarized cell layers under the influence of the extracellular matrix (ECM), and attachment to the ECM is required for the control of normal epithelial cell proliferation, differentiation, and survival [10]. The processes of proliferation and survival of malignant cells are not well recapitulated in two-dimensional (2D) cell culture. Three-dimensional (3D) cell culture models provide culture conditions that more closely mimic the in vivo environment and are used widely in epithelial cancer research to probe the mechanisms involved in tumor initiation and progression [10–12].

We examined the compensatory adaptive response of ovarian cancer cells against paclitaxel in 3D cell culture and evaluated whether inhibition of the compensatory adaptive response could increase the efficiency of paclitaxel in reducing the viability of cancer cells.

## Materials and Methods

### Cell culture

HeyA8 and SKOV3 are ovarian cancer cell lines. SKOV3 cells were obtained from the American Type Culture Collection (Manassas, VA, USA). We also obtained HeyA8 cells from Dr. Gordon Mills of the Department of Systems Biology, MD Anderson Cancer Center, Houston, TX, USA. The HeyA8 cells were derived from a human ovarian cancer xenograft (HX-62) that was originally grown from a peritoneal deposit of a patient with moderately differentiated papillary cystadenocarcinoma of the ovary [13].

**Fig 1:**
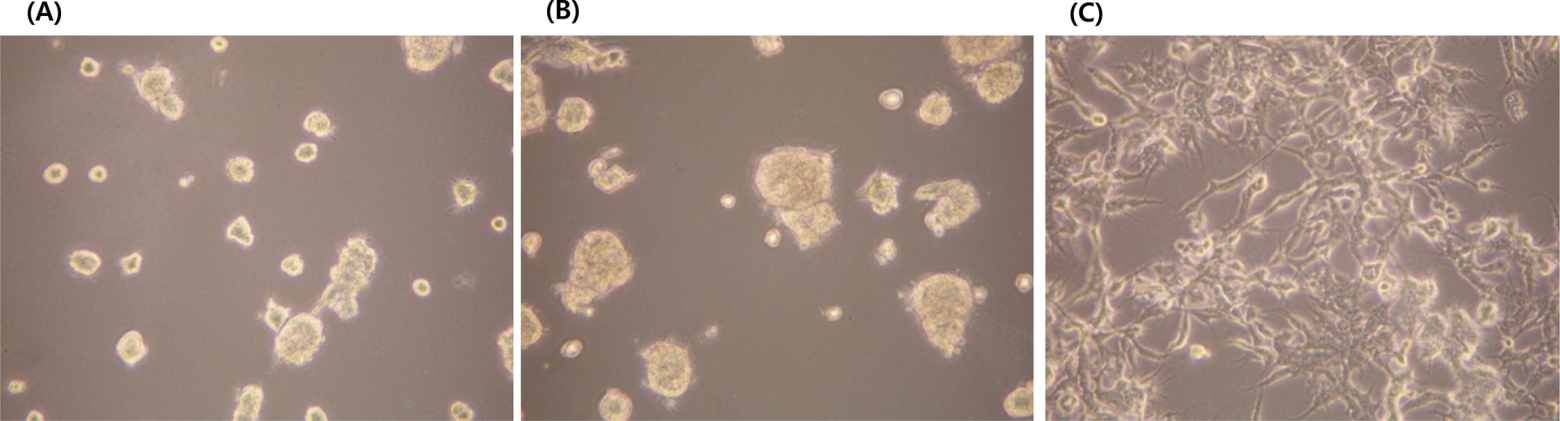
**Confluence of three-dimensional structures after 4 days of incubation of 1 × 10^5^ SKOV3 cells (A), 7 days of incubation of 1 × 10^5^ SKOV3 cells (B), and 4 days of incubation of 1 × 10^4^ HeyA8 cells (C) (100× magnification).**

The two cell lines were maintained in RPMI1640 medium (HyClone, UT, USA) containing 10% FBS (HyClone) and an antimycotic (Gibco, NY, USA) in a humidified atmosphere of 5% CO_2_. For the 3D culture, we coated each well of a 96-well plate or 12-well plate with thawed Matrigel (Growth Factor Reduced Matrigel, Corning, MA, USA) and seeded ovarian cancer cells into each well. Ten thousand HeyA8 cells or 1 × 10^5^ SKOV3 cells were seeded in a 12-well plate coated with Matrigel, and the 3D structures attained 80% confluence after 4 days of incubation (Fig 1). The same number of HeyA8 cells or SKOV3 cells were seeded in a 12-well plate for reverse-phase protein array (RPPA) or western blot analysis, and 1 × 10^3^ cells or 1 × 10^4^ cells were seeded into a 96-well plate for the cell viability assay. Paclitaxel (Selleck Chemicals, TX, USA) was added to each cell culture on day 4 for western blot analysis and the cell viability assay, and on day 6 for the RPPA. The plates were placed inside an incubator at 37°C for 24 hours for western blot analysis and the cell viability assay, and for 48 hours for the RPPA. Inhibitors of proteins involved in the compensatory adaptive response were added together with paclitaxel to HeyA8 or SKOV3 cells at the same times. The inhibitors were BX795 and CCT128930 (both from Selleck Chemicals) for inhibition of phospho-S6 kinase (pS6) (Ser240/244), pS6 (Ser235/236), and phospho-PRAS40 (pPRAS40) (Thr246).

### Cell viability assay

Cell viability was assessed using a cell counting kit (CCK-8; Dojindo Laboratories, Kumamoto, Japan) following the manufacturer’s instructions. The culture medium was removed, and 100 μL of fresh medium containing 10 μL of CCK-8 solution was added to each well. The cells were then incubated at 37°C for 4 hours. The optimal density values were determined in triplicate at 450 nm. We evaluated cell viability in HeyA8 and SKOV3 cells after treatment with paclitaxel alone; with one inhibitor of pS6 (S240/S244), pS6 (S235/S236), and pPRAS40 (T246); and with the combination of paclitaxel and an inhibitor.

### RPPA

We used RPPA to identify proteins involved in the compensatory adaptive response induced by paclitaxel in ovarian cancer cell lines. The RPPA technology is a high-throughput automated method that enables assessment of the levels of proteins and phosphoproteins across multiple sample conditions using more than 200 validated antibodies. We used paclitaxel at a concentration of 8 μM (low dose) or 10 μM (high dose) for SKOV3 cells and 0.004 μM (low dose) or 0.008 μM (high dose) in HeyA8 cells on day 6. All tests were performed in duplicate. After dissolving Matrigel by adding 800 μL of 1× Hank’s balanced salt solution with 5 mM ethylenediaminetetraacetic acid to each well, the cells were lysed by mixing 30–100 μL of lysis buffer with the cell pellet followed by centrifugation for 10 minutes. The protein concentration was measured using the bicinchoninate method. Ten microliters of 4× sodium dodecyl sulfate (SDS) sample buffer was added to 30 μL of supernatant and heated to 95°C for 5 minutes. Protein lysates were adjusted to 1 μg/μL, and a serial dilution of five concentrations was printed, with 10% of the samples replicated for quality control (2470 Arrayer; Aushon Biosystems, MA, USA) on nitrocellulose-coated slides (Grace Bio-Labs, OR, USA). Immunostaining was performed using a DakoCytomation-catalyzed system and diaminobenzidine colorimetric reaction. Spot intensities were analyzed and quantified using MicroVigene software (VigeneTech Inc., MA, USA) to generate spot signal intensities. Data from the RPPA were rearranged by the rank sum score method and are expressed in a heat map in which green indicates downregulation and red indicates upregulation of protein expression.

### Western blot analysis

Cell lysates were prepared from ovarian cancer cells in RIPA lysis buffer. Protein concentration was determined using the Lowry method (Bio-Rad, Hercules, CA, USA). Protein samples were separated using 10% SDS-polyacrylamide gel electrophoresis and transferred to a polyvinylidene difluoride membrane (Amersham Pharmacia Biotech, NJ, USA). The membrane was preincubated with 5% skim milk in Tris-buffered saline (TBS) for 1 hour at room temperature. Primary antibodies against pS6 (S240/S244) (#4838), pS6 (S235/S236) (#4858), pPRAS40 (T246) (#13175), and β-actin (Cell Signaling Technology, MA, USA) were diluted 1:1000 in TBS with 0.1% Tween 20 (TBST) and were added. The membrane was incubated overnight at 4°C and then washed three times with TBST. Horseradish peroxidase-conjugated secondary antibodies were added, and the membrane was incubated for 1 hour at room temperature. The membrane was washed in TBST, and the hybridized bands were detected using an ECL clarity detection kit (Bio-Rad) and ChemiDoc XR analyzer software Image Lab 5.1 (Bio-Rad).

### Statistical analysis

One-way analysis of variance was applied to evaluate the differences in cell viability. Significance was defined as *p* < 0.05. All statistical analyses were performed using the SPSS software package (version 20; IBM Corp., Armonk, NY, USA).

### Institutional Review Board

Approval for this study was obtained from the Institutional Review Board of the Catholic University of Korea, Bucheon, Korea.

## Results

**Fig 2:**
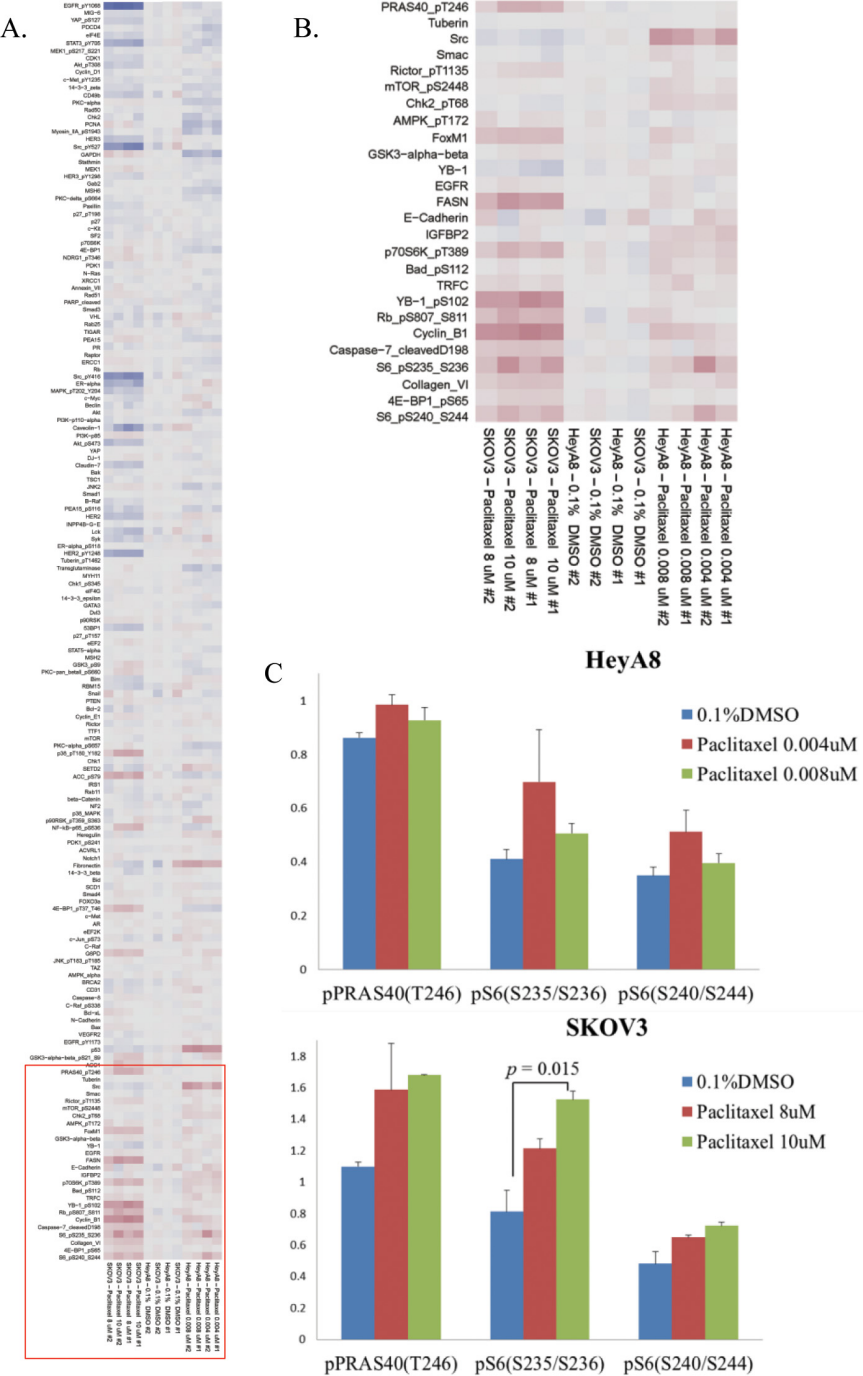
Reverse-phase protein array of HeyA8 and SKOV3 cells. (A) HeyA8 and SKOV3 cells were treated with paclitaxel in three-dimensional cell culture. The concentration of paclitaxel for each cell line are displayed on the bottom of the heat map, and the analyzed proteins on the left (blue = downregulated; red = upregulated). The red highlighted area is zoomed in B. (B) Zoomed area corresponding to the paclitaxel-specific upregulated targets. (C) Posttreatment expression levels of proteins in lysates are shown in bar graphs. *p* values were calculated using one-way analysis of variance.

**Fig 3:**
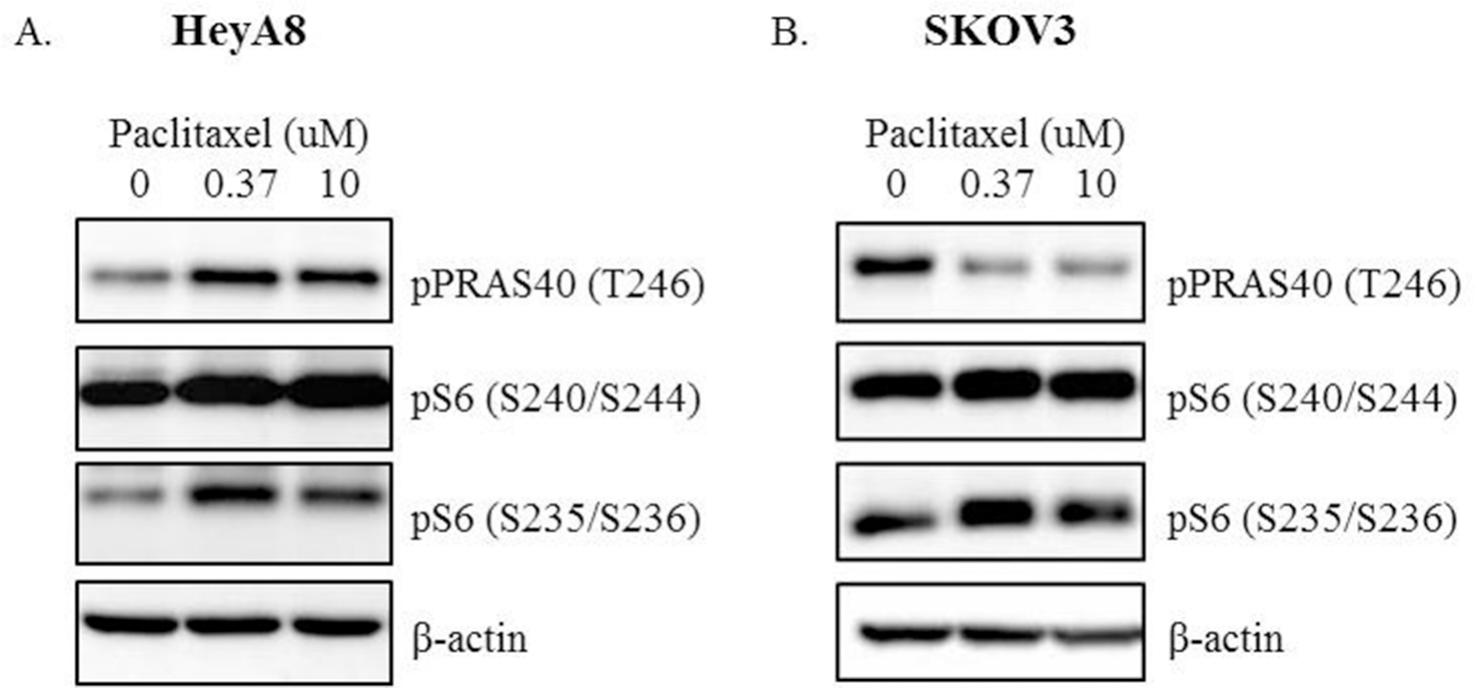
**Western blot analysis of pPRAS40 (T246), pS6 (S240/S244), and pS6 (S235/S236) proteins in (A) HeyA8 cells and (B) SKOV3 cells treated with 0.37 μM or 10 μM paclitaxel in three-dimensional cell culture for 24 hours.**

RPPA was used to identify the proteins involved in the compensatory adaptive response induced by paclitaxel in HeyA8 and SKOV3 cells (Fig 2). We evaluated >150 antibodies using RPPA. Paclitaxel induced the upregulation of pS6 (S240/S244), pS6 (S235/S236), and pPRAS40 (T246) in HeyA8 and SKOV3 cells. Western blot analysis confirmed the paclitaxel-induced increases in the expression of pS6 (S240/S244) and pS6 (S235/S236) in both cell lines (Fig 3). However, pPRAS40 (T246) expression increased only in HeyA8 cells, but not in SKOV3 cells.

**Fig 4:**
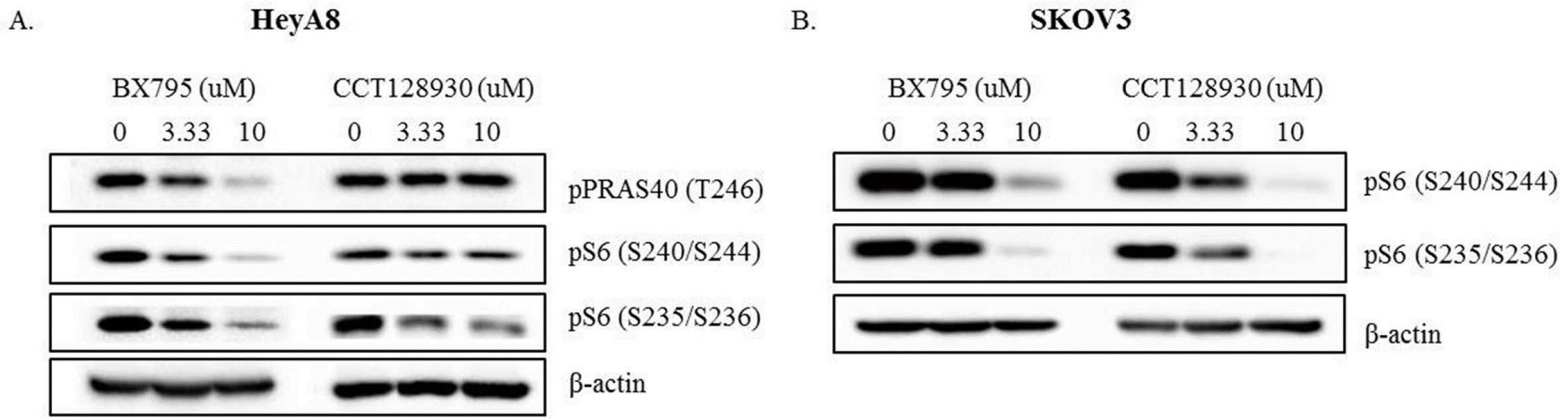
**Western blot analysis of pPRAS40 (T246), pS6 (S240/S244), and pS6 (S235/S236) proteins in (A) HeyA8 cells and (B) SKOV3 cells treated with 3.33 μM or 10 μM inhibitors (BX795 or CCT128930) in three-dimensional cell culture for 24 hours.** Ten percent fetal bovine serum was used in A and B.

We applied BX795 or CCT128930 at concentrations of 3.33 μM and 10 μM in HeyA8 and SKOV3 cells to confirm their ability to inhibit pS6 (S240/S244), pS6 (S235/S236), and pPRAS40 (T246) expression. After the 24-hour incubation, western blot analysis was performed (Fig 4). The higher concentration of BX795 and CCT128930 induced stronger inhibition of pS6 (S240/S244) and pS6 (S235/S236) expression in HeyA8 and SKOV3 cells. However, pPRAS40 (T246) expression was inhibited only by BX795, but not by CCT128930 in HeyA8 cells.

**Fig 5:**
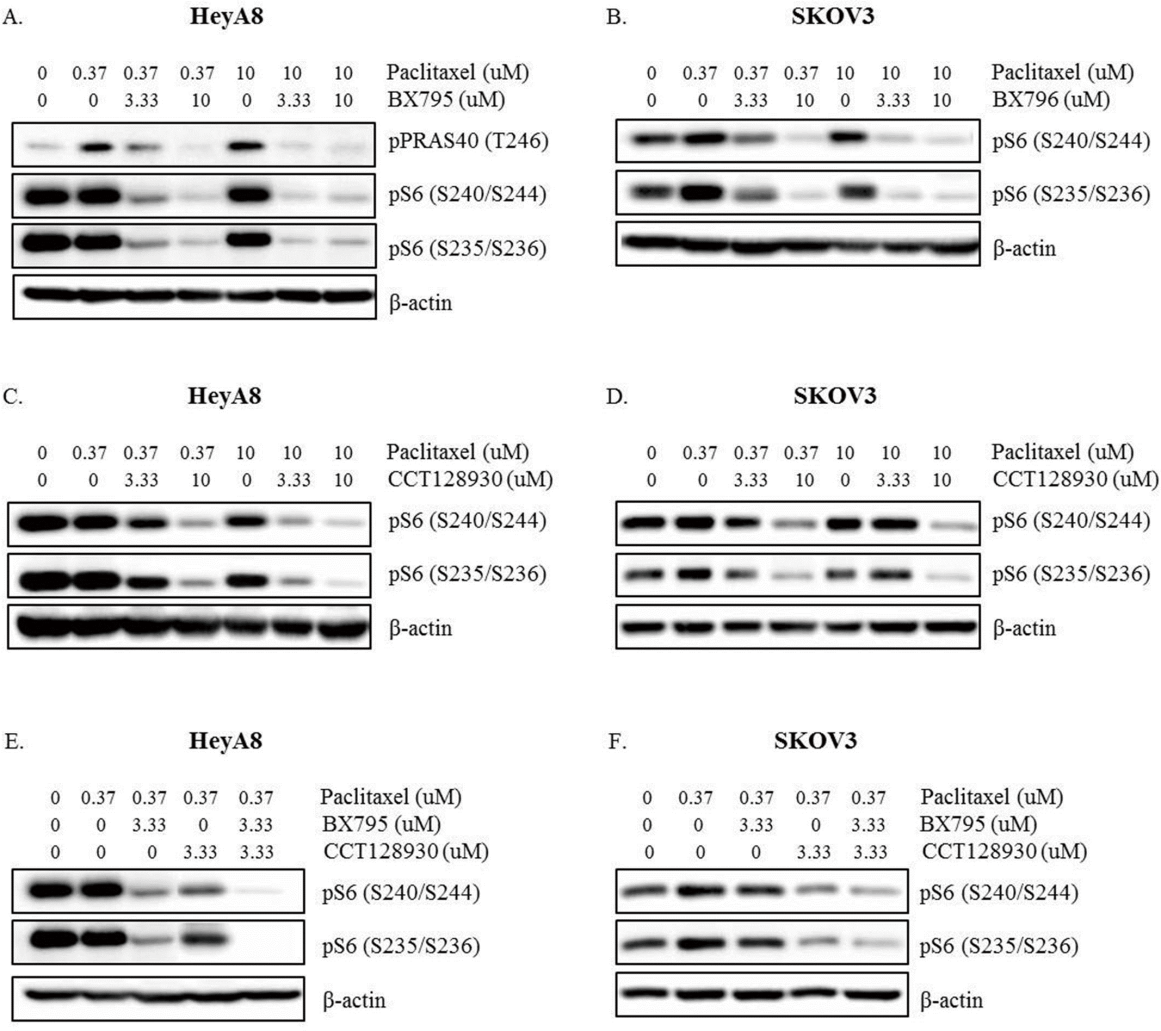
**Western blot analysis of pPRAS40 (T246), pS6 (S240/S244), and pS6 (S235/S236) proteins in (A), (C), (E) HeyA8 cells and (B), (D), (F) SKOV3 cells treated with 3.33 μM or 10 μM inhibitors (BX795 or CCT128930) combined with paclitaxel in three-dimensional cell culture for 24 hours.** Ten percent fetal bovine serum was used in (A), (B), (C), (D), (E) and (F).

The combination of paclitaxel with BX795 or CCT128930 attenuated the upregulation of pS6 (S240/S244) and pS6 (S235/S236) induced by paclitaxel in HeyA8 and SKOV3 cells (Fig 5). The combination of paclitaxel and BX795 in HeyA8 cells also decreased pPRAS40 (T246) expression. A higher concentration of inhibitor induced stronger inhibition of the expression of these proteins. Addition of the combination of 3.33 μM BX795 and 3.33 μM CCT128930 to 0.37 μM paclitaxel induced a stronger inhibition of pS6 (S240/S244) and pS6 (S235/S236) in HeyA8 and SKOV3 cells compared with addition of only one of BX795 or CCT128930 to paclitaxel at the same concentration (Fig 5E and 5F).

**Fig 6:**
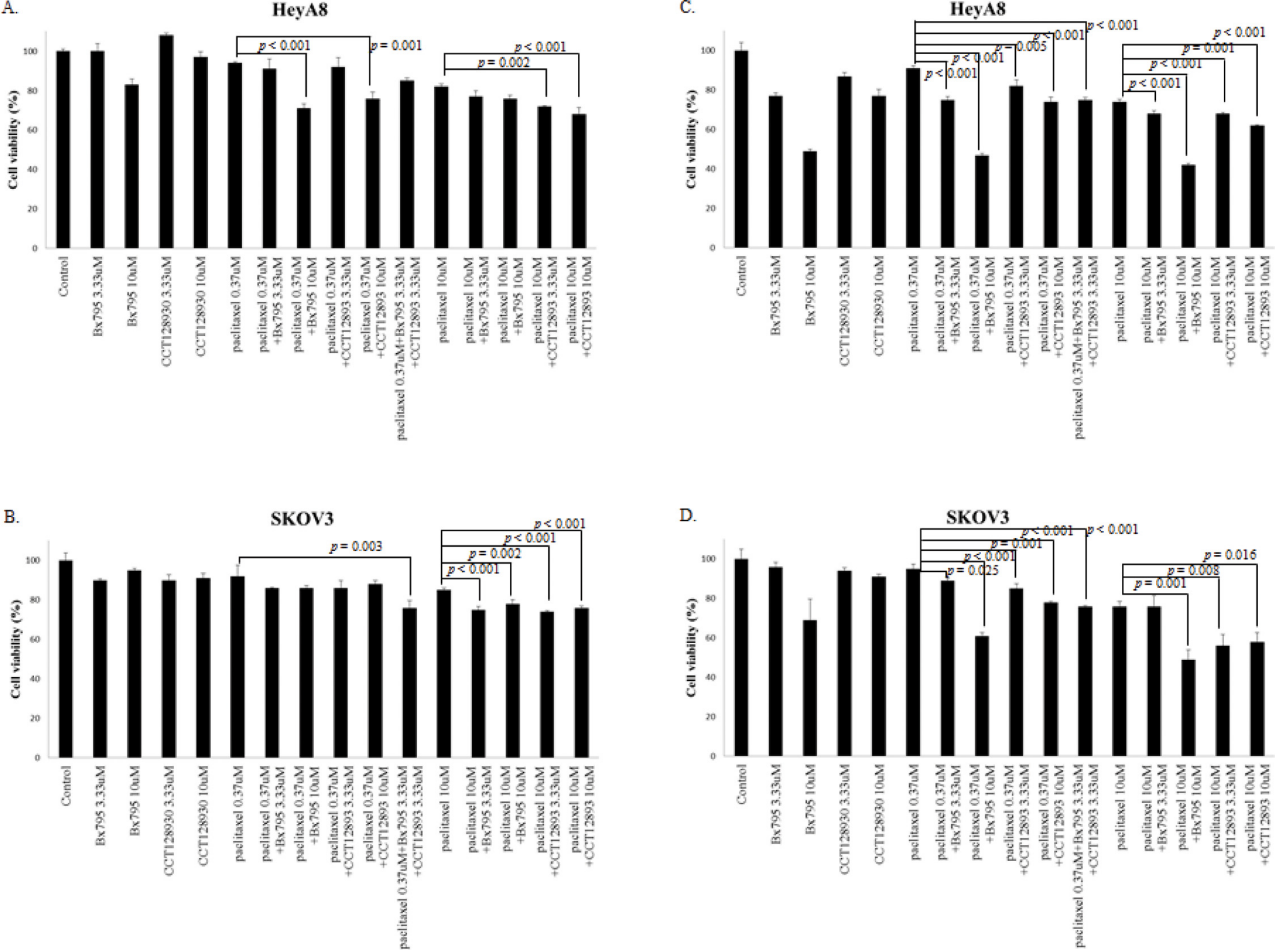
Viability of HeyA8 and SKOV3 cells after treatment with paclitaxel or inhibitors (BX795 or CCT128930) in three-dimensional cell culture for 24 hours. Ten percent fetal bovine serum was used in A and B and 1% fetal bovine serum was used in C and D. *p* values were calculated using one-way analysis of variance.

There was a trend toward a greater reduction in cell viability of HeyA8 and SKOV3 cells by addition of BX795 or CCT128930 to paclitaxel compared with paclitaxel alone (Fig 6). Addition of 10 μM BX795 or 10 μM CCT128930 to 0.37 μM paclitaxel caused a significantly greater reduction of HeyA8 cell viability compared with 0.37 μM paclitaxel alone (*p* < 0.001 and *p* = 0.001, respectively). Addition of 3.33 μM or 10 μM CCT128930 to 10 μM paclitaxel was more effective in decreasing cell viability compared with 10 μM paclitaxel alone in HeyA8 cells (*p* = 0.002 and *p* < 0.001, respectively) and in SKOV3 cells (*p* < 0.001 and *p* < 0.001, respectively). Addition of 3.33 μM or 10 μM BX795 to 10 μM paclitaxel also caused greater growth inhibition compared with 10 μM paclitaxel alone in SKOV3 cells (*p* < 0.001 and *p* = 0.002, respectively). Addition of the combination of 3.33 μM BX795 and 3.33 μM CCT128930 to 0.37 μM paclitaxel decreased cell viability of SKOV3 cells more than addition of 0.37 μM paclitaxel alone (*p* = 0.003). Addition of the combination of 3.33 μM BX795 and 3.33 μM CCT128930 to 0.37 μM paclitaxel also caused a greater decrease in cell viability compared with addition of only one of 3.33 μM BX795 or 3.33 μM CCT12893 to 0.37 μM paclitaxel in SKOV3 cells (*p* = 0.022 and *p* = 0.02, respectively). When 1% FBS was used instead of 10% FBS, the addition of 3.33 μM BX795 or 3.33 μM BX795 and 3.33 μM CCT128930 to HeyA8 cells and of 10 μM BX795 to SKOV3 cells caused a significantly greater reduction of cell viability compared with 0.37 μM paclitaxel alone (*p* < 0.001, *p* < 0.001, and *p* < 0.001, respectively)

Cell-cycle distribution was monitored by flow cytometry after treatment with paclitaxel and BX795 or CCT128930. Distribution in the sub-G1 phase, which represents the fraction of the apoptotic cell population, was increased (S1 Fig).

## Discussion

Compensatory changes in the signaling pathways of treated cancer cells can bypass drug-mediated inhibition. These changes can restore the signaling pathway activity inhibited by the targeted drug or can activate signaling components that elicit the same consequences as the original pathway. Even if an inhibitor suppresses an essential oncogenic kinase, tumor cells can exploit the interconnections between signaling pathways, switch their signaling network, and develop drug resistance [14]. Turke et al. showed inhibition of the epidermal growth factor receptor (EGFR) in non-small-cell lung carcinoma could be compensated by the increased expression of the MET receptor [15]. MET amplification activates human epidermal growth factor3 (ERBB3)–phosphatidylinositol 3 kinase (PI3K)–protein kinase B (AKT) signaling in EGFR mutant lung cancers and causes resistance to EGFR kinase inhibitors. Resistance to EGFR kinase inhibition can be cured by combined inhibition of EGFR and MET. Britschgi et al. reported that the JAK2–STAT5-evoked positive feedback loop could dampen the efficacy of PI3K–mTOR inhibition [16]. PI3K–mTOR inhibition has potent antitumor consequences. PI3K–mTOR inhibition coincides with the activation of JAK2, which in turn phosphorylates and activates STAT5, and STAT5 transactivates its target gene IL-8. The secretion and autocrine activation of the IL-8 receptor compensates for the inhibition of PI3K–mTOR.

In our study, treatment of HeyA8 and SKOV3 cells with paclitaxel increased the expression of pS6, an indicator of the adaptive response. Inhibition of pS6 (S240/S244) or pS6 (S235/S236) augmented the effect of paclitaxel in HeyA8 and SKOV3 cells. Ribosomal S6 kinases comprise a family of protein kinases involved in signal transduction. There are two known mammalian homologues of S6 kinase: S6 kinase 1 and S6 kinase 2. Both of these isoforms are also overexpressed in human cancer [2–4]. S6 kinases 1 and 2 are thought to play a tumor-promoting role in various cancers. Treatment with an S6 kinase inhibitor or siRNA against S6 kinases 1 and 2 inhibits the proliferation of prostate and breast cancer cells [2, 3], which suggests that S6 kinases 1 and 2 positively regulate cancer cell proliferation [17].

S6 kinases 1 and 2 have also been shown to positively regulate cell survival through transcription-dependent mechanisms [18]. Raf-1–extracellular signal-regulated kinase (ERK)–S6 kinase signaling has been shown to promote cellular resistance to anticancer drugs including taxanes in various cancers [19, 20]. In our study, pPRAS40 (T246) expression increased in HeyA8 cells after paclitaxel treatment, but not in SKOV3 cells. Therefore, pPRAS40 (T246) seems to be protein that is involved in the compensatory mechanisms induced by paclitaxel in HeyA8 ovarian cancer cells. We think paclitaxel inhibits only PRAS40 and not Ps6 in SKOV3 cells. HeyA8 and SKOV3 ovarian cancer cells are originated from different site and the expression patterns of proteins in ovarian cancer cells with different origins may be different. PRAS40, a direct substrate of AKT, is also a component and substrate of mTOR complex 1 (mTORC1) [21]. Thr246 of PRAS40 is an AKT consensus phosphorylation site as well as a binding site of 14-3-3 protein [21, 22]. Several stimuli including insulin, nerve growth factor, and platelet-derived growth factor increase PRAS40 phosphorylation [22, 23]. S6 is phosphorylated at Ser235/236 by p70S6 kinase. PRAS40 is required for mTORC1 signaling to p70S6 kinase, which, in turn, enables p70S6 kinase to phosphorylate S6 at Ser235/236. Although several studies have suggested that PRAS40 plays some role in cancer development, no study has shown its functional significance in tumorigenesis [24]. Using both univariate and multivariate analyses, Shipitsin et al. showed that the expression of pS6 (S235/S236) and pPRAS40 (T246) is a predictor of a lethal outcome of prostate cancer [25]. Malla et al. reported that PRAS40 is overexpressed in breast, melanoma, colon, prostate, liver, and lung cancers [26]. In contrast with our result, Kim et al. showed that paclitaxel suppresses pAKT/S6K1 in ovarian cancer cells [27]. Those authors used 20 μM paclitaxel, which is a higher concentration than used here (0.37 and 10 μM). They also performed experiments in a 2D culture setting. These differences may explain the disparity in results observed. However, Xu et al. revealed that paclitaxel increased pAKT, pS6K expression in colorectal cancer cells [28]. The HeyA8 cell line was derived from a human ovarian cancer xenograft (HX-62) that was originally grown from a peritoneal deposit of a patient with moderately differentiated papillary cystadenocarcinoma of the ovary. The manual of the American Type Culture Collection (ATCC) states that the SKOV3 cell line was derived from ascites of a patient with adenocarcinoma of the ovary. Although Beaufort et al. reported that the putative histology of SKOV3 was an endometrioid or clear cell type, the original histology is also a serous type [29]. Most patients with epithelial ovarian cancer exhibit a serous cell type. Many patients with ovarian cancer have ascites. Cells in ascites are detached from the ovarian cancer mass and float in fluid. We think that the cells present in ascites may be different from the cells found in the ovarian cancer mass regarding characteristics such as signal pathway and mutation. Therefore, we chose two ovarian cancer cells with different origins, as the expression patterns of proteins in ovarian cancer cells with different origins may be different.

RPPA is used mainly as a screening method to identify the expression pattern of proteins in cells, and treatment with drugs at low concentration is generally used for monitoring. We checked approximately the expression pattern of proteins in HeyA8 and SKOV3 cells using RPPA. After selection of candidate proteins, we confirmed expression patterns by western blotting. However, when we treated HeyA8 cells with 0.004 and 0.008 μM paclitaxel, western blotting revealed little change in the expression pattern of pPRAS40 (T246). Thus, we used 0.37 and 10 μM paclitaxel in western blotting, which clarified the expression pattern of pPRAS40 (T246). And the expression pattern of pPRAS40 (T246) was different between RPPA and western blotting in SKOV3 cells. Increased expression pattern of pPRAS40 (T246) was recorded by RPPA, whereas decrease was detected by western blotting. The methods of detection of proteins in western blotting and RPPA are different. Therefore, the results obtained via western blotting and RPPA may be different. After the use of RPPA as a screening method, western blotting is usually needed as confirmation. Sometimes, because the pattern of color change in a heat map may be different from the real pattern of expression of a protein, double check by bar graph should be performed simultaneously. However, the difference in the expression pattern of pPRAS40 (T246) between RPPA and western blotting in SKOV3 cells was really big. It is possible that there are other reasons but unfortunately we failed to find out the reasons.

To inhibit pS6 (S240/S244) and pS6 (S235/S236) in HeyA8 and SKOV3 cells, we tried AT7867, BX795, and CCT128930. However, AT7867, which is described by the manufacturer (Selleck Chemicals) as an AKT inhibitor, did not inhibit the expression of pS6 (S240/S244) or pS6 (S235/S236) at concentrations of 0.37, 1.11, and 3.33 μM in HeyA8 and SKOV3 cells (data not shown). BX795 is described by the manufacturer as an inhibitor of TANK-binding kinase 1 (TBK1) and of IκB kinase ε (IKKε), and CCT128930 is a known AKT2 inhibitor. Vu et al. reported that TBK1 depletion inhibits migration and invasion, whereas its overexpression increases the invasive ability of melanoma cells [30]. They also confirmed that BX795 in combination with AZD6244, an MEK inhibitor, increases apoptosis in AZD6244-resistant melanoma cells. Wang et al. showed that CCT128930 increases the phosphorylation of AKT in HepG2 hepatoma cancer cells and that CCT128930 inhibits cell proliferation by inducing cell cycle arrest in G1 phase [31]. However, we found that BX795 did not inhibit the expression of pTBK1 (S172) and that CCT128930 did not inhibit the expression of pAKT (S473) at concentrations of 3.33 and 10 μM in HeyA8 and SKOV3 cells (S2 Fig). We estimate that CCT128930 inhibits only pS6 (and not pRAS40 and pAKT), and that BX795 inhibits the mTOR pathway rather than TBK1, at concentrations of 3.33 and 10 μM. In our study, BX795 and CCT128930 at 3.33 μM or 10 μM inhibited the expression of pS6 (S240/S244) and pS6 (S235/S236) in HeyA8 and SKOV3 cells, and the inhibition was greatest at the higher concentration. We could not find other studies of the use of BX795 or CCT128930 to inhibit pS6 (S240/S244) and pS6 (S235/S236). BX795 also inhibited pPRAS40 (T246), but CCT128930 did not inhibit pPRAS40 (T246) in HeyA8 cells. We presume that BX795 inhibits the PRAS40–S6 signaling pathway and that CCT128930 inhibits pS6 via the inhibition of other signaling pathways not including PRAS40 or inhibits pS6 directly. Therefore, there is not any evidence that TBK1 is associated with PI3K/AKT/mTOR pathway in our study.

Fig 5A of our study shows that cotreatment with BX795 (3.33 and 10 μM) and paclitaxel (10 μM) caused a significant decrease in the expression of pPRAS40(T246), pS6(S240/S244), and pS6(S235/S236). However, Fig 6A shows that the same treatment did not yield a significant inhibition of cell viability in HeyA8 cells. A similar response is observed in Fig 5E, as cotreatment with the three drugs reduced the expression of pS6(S240/S244) and pS6(S235/S236), but did not inhibit cell viability significantly, as shown in Fig 6A. When 1% FBS was used instead of 10% FBS, the addition of BX795 or CCT128930 to HeyA8 cells and SKOV3 cells caused a significantly greater reduction of cell viability compared with paclitaxel alone (Fig 6C and 6D). The 3D cell culture creates an artificial environment in which biological cells are permitted to grow or interact with their surroundings in three dimensions. Thus, the 3D model mimics more accurately the in vivo condition and cell functions compared with 2D cell culture, which lacks oxygen, nutrient, and waste gradients, and the environment is not physiologically uniform. 3D cell culture can provide a more accurate depiction of cell polarization and cells grown in 3D culture have greater stability and a longer lifespan than cells grown in 2D culture [32]. 3D aggregates can be cultured for up to 4 weeks compared with nearly 1 week in 2D culture because the cells reach confluency. We also cultured cells in the 3D model using Matrigel for more detailed experiments. The shapes of the SKOV3 cells differ between the 3D and 2D cultures. However, HeyA8 cells grow in a similar pattern in 2D and 3D culture. In the study reported by Nanos-Webb et al., the growth pattern of HeyA8 cells in two culture environments was also similar [33]. We performed many experiments to determine the optimum conditions for adequate confluence of cells in the 3D cultures. When we seeded 2 × 10^4^ HeyA8 cells in a 12-well plate, the cells reached a too-complex confluence before the adequate formation of a 3D structure. Ten thousand HeyA8 cells filled about 80% of 12-well plate space and reached an adequate 3D structure under a microscope on day 4 of incubation. We defined this condition as adequate confluence for HeyA8 cells. As SKOV3 cells grew more slowly than HeyA8 cells, we seeded 8 × 10^3^ cells in a 12-well plate. SKOV3 cells divided and grew for 4 days, but after that time, cells stopped dividing and only grew, i.e., the size of cells increased but their number did not. Therefore, we performed experiments on day 4 of SKOV3 cell incubation.

The expression of pS6 (S240/S244) and pS6 (S235/S236) increased in HeyA8 and SKOV3 ovarian cancer cells, and the expression of pPRAS40 (T246) increased in HeyA8 cells after exposure to paclitaxel. These effects are consistent with the compensatory adaptive response after treatment of paclitaxel. Adding BX795 or CCT128930 as an inhibitor of pS6 (S240/S244) and pS6 (S235/S236) in HeyA8 and SKOV3 cells, and adding BX795 as an inhibitor of pPRAS40 (T246) in HeyA8 cells augmented the effects of paclitaxel in decreasing cell viability compared with addition of paclitaxel alone.

## Supporting Information

S1 Fig Cell-cycle distribution was monitored by flow cytometry after treatment with paclitaxel and BX795 or CCT128930 in three-dimensional cell culture for 24 hours. Distribution in the sub-G1 phase represents the fraction of the apoptotic cell population.
(TIF)

S2 Fig **Western blot analysis of the pTBK1 (S172) and pAKT (S473) proteins in HeyA8 and SKOV3 cells treated with 3.33 and 10 μM BX795 (A) or CCT128930 (B) in three-dimensional cell culture for 24 h.**
(TIF)
