# Supplementary figures and images for: Inhibition of Phospho-S6 Kinase, a Protein Involved in the Compensatory Adaptive Response, Increases the Efficacy of Paclitaxel in Reducing the Viability of Matrix-Attached Ovarian Cancer Cells
Source: PLoS One. 2016 May 5;11(5):e0155052. doi: 10.1371/journal.pone.0155052 (PMC4858236; doi:10.1371/journal.pone.0155052)

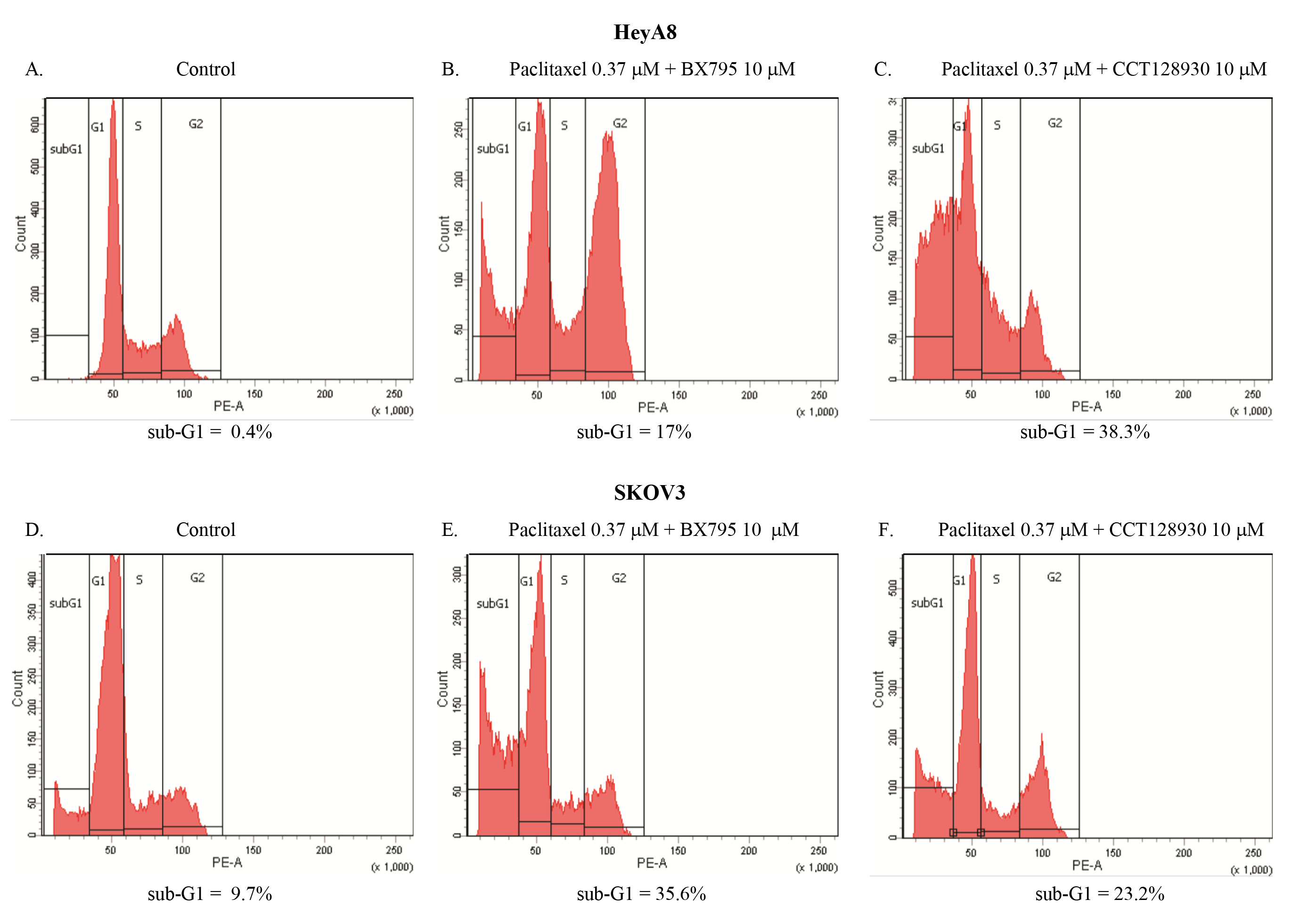

Supplement: S1 Fig — Distribution in the sub-G1 phase represents the fraction of the apoptotic cell population. (TIF) [file pone.0155052.s001.tif]

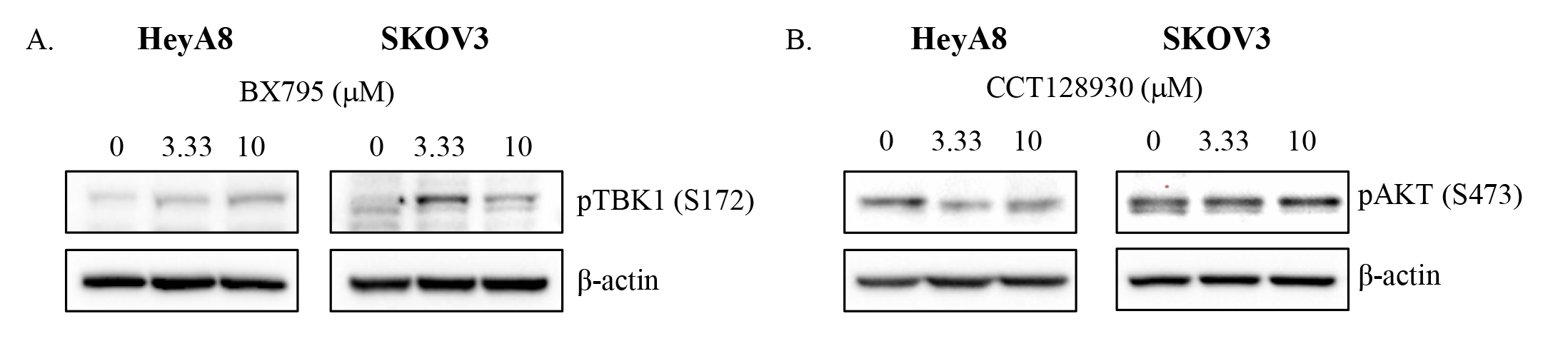

Supplement: S2 Fig — Western blot analysis of the pTBK1 (S172) and pAKT (S473) proteins in HeyA8 and SKOV3 cells treated with 3.33 and 10 μM BX795 (A) or CCT128930 (B) in three-dimensional cell culture for 24 h. (TIF) [file pone.0155052.s002.tif]
